# Purpose in Life and Cognition: Momentary Associations From a Micro-Longitudinal Study of Daily Life

**DOI:** 10.1093/geroni/igaf122.464

**Published:** 2025-12-31

**Authors:** Angelina R Sutin, Martina Luchetti, Antonio Terracciano

**Affiliations:** Florida State University College of Medicine, Tallahassee, Florida, United States; Florida State University College of Medicine, Tallahassee, Florida, United States; Florida State University, Tallahassee, Florida, United States

## Abstract

Purpose in life, the feeling the one’s life is goal-oriented and has direction, is associated with healthier cognitive outcomes in older adulthood. A better understanding of how purpose supports cognitive function in daily life is needed to inform intervention development. The present study replicates and extends recent evidence that momentary feelings of purpose help support cognitive function throughout the day. Using ecological momentary assessment methodology, participants from the Purpose and Engagement in Everyday Life study (N = 1573; Mage=66.06, range 40-93) reported on their momentary purpose and cognitive complaints and completed three short cognitive tasks three times a day for eight days (n = 32,375 momentary assessments). Consistent with the literature, between-person, momentary purpose was associated with better performance on the three cognitive tasks and reporting fewer cognitive complaints. Within person, in moments when participants felt more purposeful than their average, they had faster processing speed (b=-.05, SE=.02, p=.002) and executive functioning (b=-.16, SE=.05, p<.001) and reported fewer problems with concentration (b=-.10, SE=.01, p<.001); momentary purpose was unrelated to visual memory. Momentary purpose, however, was also associated with more reported memory lapses measured concurrently in the moment (b=.03, SE=.01, p=.002) and at the next moment (b=.04, SE=.02, p=.005). The present findings replicate the momentary association between purpose and processing speed with different measures of both constructs than used in previous research, extends the momentary association to executive functioning and concentration, and suggests that purpose may generally be beneficial, but it may tax attentional resources that temporarily results in forgetfulness in daily life.

